# Classification and Characterization of Traumatic Brain Injuries in the Northern Region of Sweden

**DOI:** 10.3390/jcm13010008

**Published:** 2023-12-19

**Authors:** Beatrice M. Magnusson, Lars-Owe D. Koskinen

**Affiliations:** 1 Department of Surgery and Perioperative Sciences, Anaesthesiology and Intensive Care Medicine, Umeå University, 901 87 Umeå, Sweden; 2 Department of Clinical Science, Neurosciences, Umeå University, 901 87 Umeå, Sweden; lars-owe.koskinen@umu.se

**Keywords:** traumatic brain injury, epidemiological, demographics, Glasgow Coma Scale, CT scan, intervention, admission, outcome, prospective

## Abstract

Background: Traumatic brain injury (TBI) is a common cause of death and disability, the incidence of which in northern Sweden is not fully investigated. This study classifies and characterize epidemiological and demographic features of TBIs in a defined population in Umeå county, Sweden. Specifically, to evaluate frequencies of (1) intracranial lesions detected with computed tomography (CT), (2) need for emergency intervention, and (3) hospital admission, in minimal, mild, moderate, and severe TBI, respectively. Methods: The data were gathered from 4057 TBI patients visiting our emergency room (ER) during a two-year period (2015–2016), of whom 56% were men and approximately 95% had minimal TBIs (Glasgow Coma Scale (GCS), score 15). Results: Of all injuries, 97.8% were mild (GCS 14–15), 1.7% were moderate (GCS 9–13), and 0.5% were severe (GCS < 9). CT scans were performed on 46% of the patients, with 28% being hospitalized. A high annual TBI incidence of 1350 cases per 100,000 citizens was found. The mortality rate was 0.5% with the majority as expected in the elderly group (>80 years). Conclusions: Minimal TBIs were not as mild as previously reported, with a relatively high frequency of abnormal CT findings and a high mortality rate. No emergency intervention was required in patients in the GCS 13–15 group with normal CT scans. These findings have implications for clinical practice in the ER with the suggestion to include biomarkers to reduce unnecessary CT scans.

## 1. Introduction

Traumatic brain injury (TBI) is considered the most complex disease in our most complex organ, with great heterogeneity regarding etiology, mechanism, severity, and treatment. TBI is defined as an alteration in brain function, or other evidence of brain pathology, caused by an external force [1]. The outcomes vary widely depending on the impact of the contusions, diffuse cellular damage, intracranial hematomas, and axonal injuries. TBI is not only an acute event, but often a chronic process, with progressive injury occurring over the course of several years [2].

TBI poses a global health challenge of vast, but insufficiently recognized, proportions [3]. It is estimated that about half the world’s population will have suffered at least one TBI in their lifetime [1]. In developed countries, it is a major cause of death and disability in young adults [4]. The annual incidence worldwide varies between 100 and 300 cases per 100,000 inhabitants, but the true number affected by TBI is much higher [5,6,7].

In high-income countries, the most common trauma source is falls. If patients are divided into age groups, traffic accidents are common in the young adult population, and fall injuries among children and the elderly [8,9]. In high-income countries, fall injuries—especially among the elderly—have increased to a greater extent, which can be attributed to demographic aging [9,10]. Furthermore, there has been an increase in TBI in the pediatric subpopulation [1,9]. Overall, men have a higher incidence of head injury than women [1,10], primarily due to high-risk behaviors [11]. In Europe, the overall mortality rate from TBI is estimated at 10.5–11.5 per 100,000 inhabitants [4,12], and in Sweden at 9.5 per 100,000 inhabitants [13].

At an acute clinical examination, TBI is usually classified from mild to severe on the Glasgow Coma Scale (GCS), based on eye opening, verbal response, and motoric reaction [14]. Concussions are seen at the milder end of the spectrum. Patients with severe injuries arrive at the emergency room (ER) unconscious. There is a strong correlation between GCS score and morbidity at the severe end of the spectrum, but a very limited correlation at the milder end [15,16]. The number of TBI patients constitutes a large group at the ER where both the characteristics of the patients and their care pathways in Europe are poorly understood [17,18,19,20]. Over 90 percent of TBI patients at the ER will have a mild injury without any life-threatening consequences, but it is of the utmost importance to identify the minority who are at risk [1]. It is very difficult for a clinician to justify a CT scan, due to the known radiation risk, and to decide when a patient can safely be sent home or needs to be admitted to hospital. Existing guidelines are generic, with a one-size-fits-all approach that is not focused on individual patient needs [21,22,23]. Evidence to support treatment guidelines and recommendations is scarce [1].

Thus, TBI is an important cause of death and disability, with great personal suffering for the victims and relatives alike and with enormous direct and indirect costs to society. For the development of new guidelines, the true incidence of consequences following TBI must be elucidated. There are only a few population-based studies on the epidemiological aspects of TBI in northern Sweden, all with small sample sizes [8,22]. None of these studies have included the total flow of TBI patients in the ER. Given the changing epidemiology seen across Europe wherein injury demographics and characteristics are changing, it is important to examine the incidence of TBI in northern Sweden, which has never been fully investigated before. The overall aim of this study was therefore to evaluate epidemiological and demographic features of TBI in patients of all ages seeking care at the ER in a defined population in Umeå county. The specific aims were to evaluate the frequencies of (1) intracranial lesions detected with CT, (2) need for emergency intervention, and (3) hospital admission, in minimal, mild, moderate, and severe TBI, respectively.

## 2. Methods

### 2.1. Design and Study Population

This study was a single-center prospective observational cohort study of TBI patients. The study population consisted of all patients who visited the ER at Umeå University Hospital (NUS) during a period of 24 months between January 2015 and December 2016. NUS is the only neurotrauma center in the North Health Region of Sweden, with a catchment area of approximately 150,000 inhabitants. The hospital also serves about 270,000 inhabitants at a county level and 900,000 inhabitants at a regional level. All patients, regardless of age and gender, with suspected TBI and who suffered an event of clear external mechanical force to the head were included. All diagnosis codes relating to TBI were used. Patients without a confirmed head trauma, transferred from other hospitals or transferred directly to the intensive care unit (ICU), were excluded.

### 2.2. Definition of Variables

In all cases, the clinical, demographic, and epidemiological data were collected prospectively at the ER. The initial management of the TBI at the ER was based on the Scandinavian Neurotrauma Committee guidelines on management of TBI in adults [21] and children [23]. The decision to perform a CT scan was made by the ER physician and thus reflected everyday clinical practice. Severe TBI was treated in accordance with a modified Lund concept [24]. Radiological assessments were performed by neuroradiologists. CT images were reviewed by at least two medical specialists, one being a senior doctor. Only variables from the first CT examination were studied. One patient with severe TBI was pronounced dead before a CT was performed. The first CT scan of the head was used for describing the eventual pathology of the brain.

Minimal TBI was defined as GCS 15, mild as GCS 14–15, moderate as 9–13, and severe as <9, respectively, based upon evaluation at ER admission. The groups GCS 13, 14, and 15 were separated for further analysis. The American Society of Anesthesiologists (ASAs) classification was used to assess each patient’s pre-injury medical comorbidities [25].

CT results were assessed as abnormal if there was any acute intracranial injury or evidence thereof: compressed basal cisterns, 0–4 mm, >5 mm, >10 mm midline shifts, epidural hematoma (EDH), acute subdural hematoma (ASDH), traumatic subarachnoid hemorrhage (tSAH), contusion (single or multiple lesions, also including intraparenchymal hemorrhage), other findings (non-traumatic or non-acute intracranial findings such as ischemia, tumors, white matter changes, and chronic subdural hematoma), and fractures (facial fractures, skull fractures, or dens fractures). Hospital admission was defined as admission to either a ward and/or an ICU. Need for emergency intervention was defined as need for intubation, craniotomy, craniectomy, ventricular drainage, or insertion of an intraparenchymal pressure (ICP) monitoring device. A patient could receive several interventions, though this was counted as one intervention. Injury type was divided into penetrating (defined as mechanical trauma to the head that penetrated the skull), blunt/closed (blunt force), and crush (defined as mechanical trauma to the head between two or more hard objects, including lacerations). Injury mechanism was divided into falls from standing height, falls from height (>1 m), bicycle accidents, car accidents, other motor vehicle and traffic accidents (e.g., motorcycles, mopeds, EPA tractors, snowmobiles, all-terrain vehicles, and pedestrians hit by a motor vehicle), assault, sports injuries, struck by or against an object (defined as either the patient being struck by a high-energy object to the head or the patient’s head being the high-energy object striking an object), and unclear (when the patient could not explain or there were no witnesses to the trauma). A distinction was made between high-energy transfer mechanisms such as traffic accidents, sports injuries, falls from height, struck by or against an object and assault, and low-energy transfer mechanisms such as falls from standing height in the horizontal plane [3]. Place injury occurred was divided into home, public place, street/traffic, sports field/horse stable (including sports hall), outside (including outdoor activities such as skiing, skateboarding, and using other motor vehicles that were not driven in traffic such as snowmobiles), work, school/kindergarten (including pre-school and playground), hospital/nursing home (including service home), others (e.g., party, nightclub, refugee accommodation, jail, unspecified garage), and unknown (when the location is unknown due to missing information in clinical records). The patients were divided into six age groups: <20, 20–29, 30–49, 50–64, 65–79, and 80–99 years. Mortality was assessed at discharge from the hospital or ER.

### 2.3. Statistical Analysis

Statistical analysis was performed using Excel for Microsoft 365 MSO (version 2310) and Statistical Package for the Social Sciences (IBM SPSS, v. 25). Categorical variables are presented as percentages. Median, interquartile range (IQR), and range were used where appropriate. A statistical significance test was performed with tests for proportions (chi-squared), non-parametrical, or parametrical tests, depending on the underlying parameters. A *p* value < 0.05 was considered statistically significant.

## 3. Results

### 3.1. Overall Cohort Characteristics

**Figure 1:**
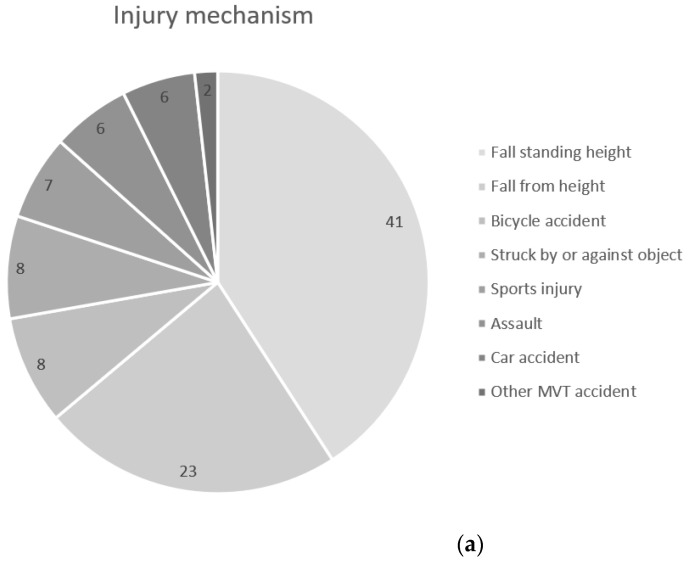

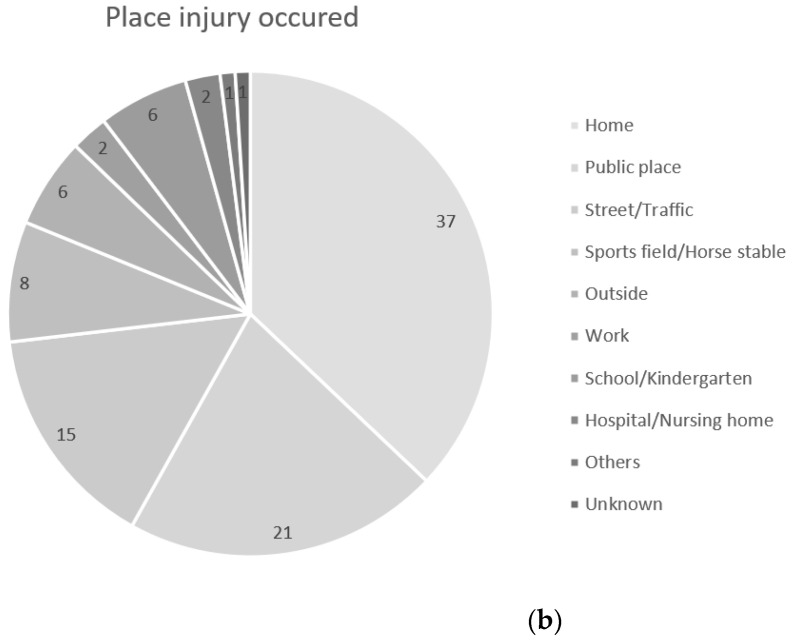
Percentage of different mechanisms (**a**) and location of injury (**b**) for the study cohort. MVT: motor vehicle and traffic accident.

The incidence of TBI at NUS is 1350 per 100,000 citizens per year. Demographic, epidemiological, and clinical characteristics of the cohort are shown in detail in Table 1 and Table 2. The average age was 37 years (median 27, IQR 11 to 63), in the range of 0–99, with 56% being men. The average age among men was 34 years (median 25, IQR 11 to 63), in the range of 0–97, and that among women was 40 years (median 32, IQR 11 to 63), in the range of 0–99. Six percent of the patients (*n* = 253) had repeat visits due to their TBIs. Upon arrival to the ER, approximately 95% of the cases were classified as minimal (GCS 15), 97.8% as mild (GCS 14–15), 1.7% as moderate (GCS 9–13), and 0.5% as severe TBI (GCS < 9), respectively. The most common injury type was blunt/closed trauma. Falls were the most common injury mechanism, followed by traffic accidents, as shown in Figure 1a. The most common place injuries occurred at was at home, followed by public places and street/traffic, as shown in Figure 1b.

**Figure 2:**
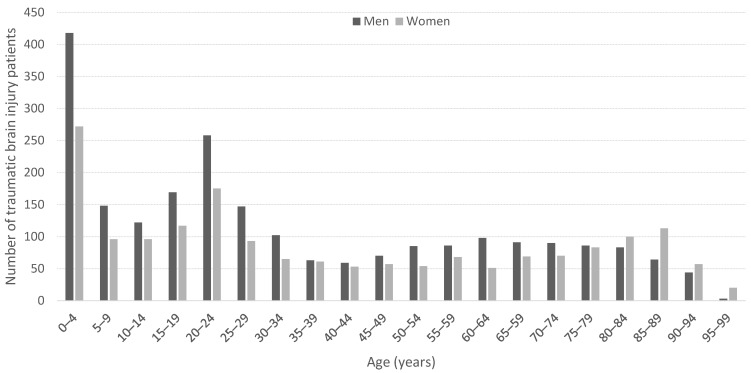
The gender and age distribution of TBI patients over a 2-year period.

Figure 2 shows the age distribution, with the highest number of TBIs in the younger age groups for both genders. In the older age groups, the proportion of injured men decreased, whereas the proportion of women increased.

### 3.2. Differences between Age Groups

Table 1 shows that the highest percentage of fall trauma was found in the age group <20 years and among the elderly >65 years. The elderly sustained most of their fall injuries from standing, whereas children and teenagers fell from greater heights. Bicycle accidents were the most common traffic accident in all age groups, except for those aged >65 years, but especially common at ages 20–64. Sports injuries accounted for many injuries in children, teenagers, and young adults aged 20–29 years, whereas assault accounted for 10–17% for the aged 20–49 years. The largest proportion of traffic accidents was found among those aged 20–49 years. Most of the TBIs for the youngest and oldest age groups occurred at home: 42% and 69%, respectively. Children and teenagers suffered most of their injuries either outdoors, on a playground or at school/kindergarten, whereas the age groups between 20 and 79 years were more exposed in street/traffic or in public places. As expected, the comorbidity based on the ASA-PS classification increased with increasing age.

**Figure 3:**
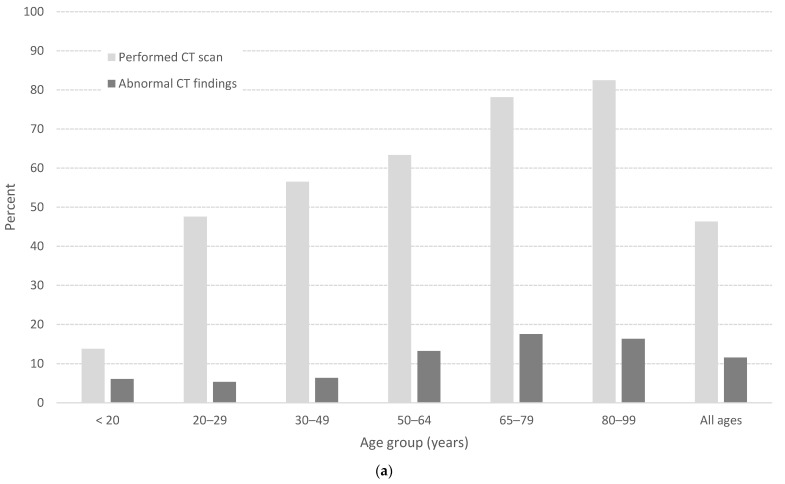

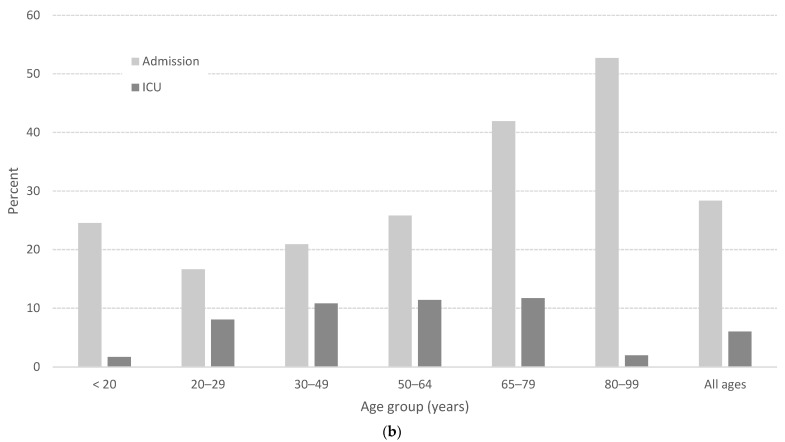
The proportion of patients, divided into age groups, with (**a**) performed computed tomography (CT) scan and abnormal CT findings, and (**b**) admission to hospital or intensive care unit (ICU).

Figure 3a shows the frequencies of CT scans and of scans with abnormal intracranial findings. Overall, CT scans were conducted on 46% of the patients, with 14% conducted on patients younger than 20 years. Among patients aged >20 years, a CT scan was carried out in 60–80% of them. Significantly more pathological changes were detected in the elderly, with 70% of all changes being seen in patients over 65 years and only 6% in the youngest age group (*p* < 0.01). Figure 3b shows a total hospitalization rate of 28%, with 6% of those hospitalized being cared for in the ICU. As expected, hospitalization was most common for the oldest age groups. Patients aged 50–79 years had the highest proportion of ICU admissions. Our results demonstrate a low mortality rate of 0.5%, with all but one case affecting elderly patients (mean age 80 years). The mortality rate was slightly higher among women than among men (0.7% versus 0.4%).

### 3.3. Differences between TBI Severities

**Figure 4:**
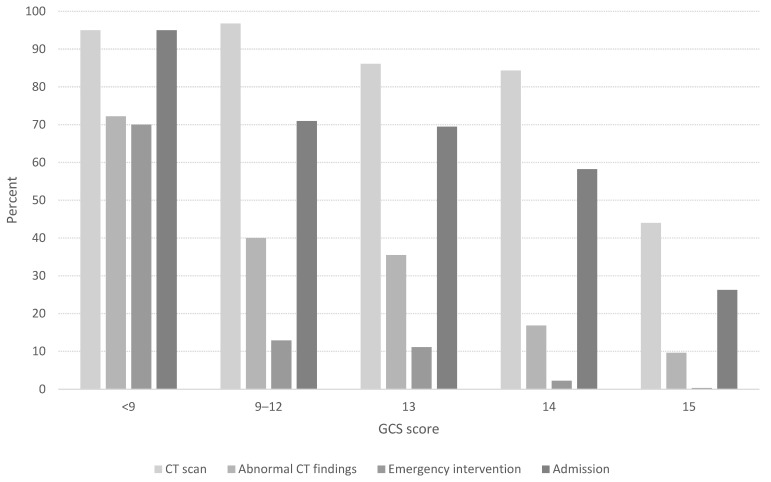
The proportions of patients with CT scans, abnormal findings, emergency interventions, and hospital admissions by Glasgow Come Scale (GCS) score.

Figure 4 shows the percentage of performed CT scans, abnormal findings, emergency interventions, and hospital admissions for different severities of TBI.

All severities had a slightly higher preponderance of men, as shown in Table 2. Most cases of severe TBI affected adults after a high-energy trauma on the street or in traffic, where the CT examination showed large intracranial changes. Many patients with severe TBIs were cared for in the ICU after undergoing some form of emergency intervention. Moderate TBI mostly affected adults aged 20–64 years, where the cause was usually a fall in the home. Almost all patients with moderate TBIs underwent a CT scan, which in 35–40% of cases revealed some form of pathology. Most of the patients with a moderate TBI were admitted to a regular ward, with approximately 10% undergoing some form of emergency intervention. In the GCS 14 group with mild injuries, patients over 50 years of age predominated, usually due to fall trauma at home or in a public place, with 84% undergoing a CT scan. Of these, 17% demonstrated some form of abnormal change. The highest proportion of children, adolescents, and young adults was found in the group with minimal TBIs, with falls at home or in public places being the most common cause of injury. In the GCS 15 group, 44% of patients underwent a CT scan, with 10% demonstrating pathological findings and 26% of those patients being hospitalized.

The most common intracranial pathological findings were tSAH, ASDH, and cerebral contusions, as shown in Table 3. Less than 1% of the patients received emergency and neurosurgical procedures, with the most common being intubation, followed by craniotomy and insertion of ICP monitoring. Patients with minimal TBIs who underwent some form of emergency intervention all exhibited pathological changes on their CT scans—83% were men. No patients in the GCS 13–15 group on whom a CT scan was performed with normal findings required any type of emergency intervention. Men had a higher frequency of abnormal CT findings and admissions, although not significantly higher. However, men had significantly higher rates of penetrating brain injury (*p* < 0.04), ICU admissions (*p* < 0.01), and emergency and neurosurgical interventions (*p* < 0.02).

The mortality rate for minimal TBIs was 0.2% (8 of 3836) compared to 40% (8 of 20) in severe TBIs assessed at discharge from the hospital. All fatal cases classified as minimal were fall traumas in elderly patients. Fifty percent of the deaths in the severe TBI group were due to traffic-related accidents and 40 percent were caused by fall trauma. Two of the patients with severe TBIs had SAH as the cause of death with uncertainty as to whether it was traumatic or spontaneously induced. All but one of the deaths among women had some degree of multimorbidity in the background. Additionally, 85% of the deaths among women occurred in the elderly group, with an average age of 87 years, having sustained the injury due to a fall in the home.

## 4. Discussion

This study classifies and characterizes injury descriptors for TBI patients in all age groups and degrees of severity in northern Sweden. To the best of our knowledge, no study of a similar selected cohort, prospectively and consecutively measured during a two-year period, has previously been reported. We demonstrate a much higher incidence of TBI than previously reported in studies at NUS [26] or other parts of Europe [4,5,7,9,12]. Annually, 2000 patients are diagnosed with TBI at NUS, which has a catchment area of about 150,000 inhabitants, suggesting an annual incidence of 1350 per 100,000 inhabitants. This is much higher than previously reported figures of 100–300 per 100,000 [5]. The annual incidence of severe TBI was 6.7 per 100,000 inhabitants, in line with previously estimated values of 4–8 per 100,000 [11]. The difference was mainly due to our high prevalence of minimal TBI, where we used a broader definition with the aim of including all patients, to illustrate the true patient flow. The difference in results may also be because some previous studies have calculated incidence based on hospital stays alone, leaving out the statistics for minimal and mild TBIs discharged directly to the home. Comparing the annual incidence based on admission alone, our results are more in line with previous studies, although still high, at about 380 per 100,000 inhabitants. We have included all data, regardless of injury severity, to illustrate true numbers. Access to Swedish healthcare can also contribute to the high numbers, as all citizens can rapidly receive a medical assessment at the ER, which is open around the clock, year-round. Another reason that should not be underestimated is our winter climate with cold temperatures and snow for a large part of the year. Many of the injuries included in this study were caused by slippery surfaces and may have occurred under the influence of alcohol and/or drugs.

The trend in Europe is that the population of TBI patients is becoming older [9,10]. Since the 1980s, the median age has nearly doubled [1]. However, our median age of 27 years is in the lower part of the spectrum compared with that in the rest of Europe [9]. This may mean that TBI patients in northern Sweden are younger than in other areas of Europe. However, we showed that patients had a higher median age than in an earlier study at NUS, which reported a median age of 23 years among men and 22 years among women [8]. In our study, the average age among women was higher than that among men. Our age distribution resembles the results of a study from the USA [27], wherein the maximum incidence was demonstrated in the age groups 0–4, 15–24, and over 75 years. However, there are differences, such as the fact that their highest TBI incidence was found among those aged over 75 years and for us the peak was among younger children. The late peak among women over the age of 80 years is also seen in other European countries [4,28].

The most common injury mechanisms were falls and traffic accidents, as previously demonstrated, though the proportion of falls was high in our study [1,8,9,27]. Children and elderly patients had the highest incidences of fall-related TBIs. The children had a high incidence of vertical falls from a certain height, whereas for the elderly, horizontal falls from standing height predominated. The younger age groups had a high frequency of being struck by or against an object, which has also been seen previously [1]. However, making comparisons using this category is difficult due to its overlap with other categories. In the young adult population, fall-related injuries were more common than traffic accidents, which contradicts some previous findings [9]. Young adults aged 20–29 years and adults aged 30–49 years had a higher proportion of traffic accidents than any other age group.

Many TBI patients have never sought care, despite having long-term residual symptoms. Some of them, especially after repeated TBIs, may acquire lifelong problems. The true incidence of sport-related concussions is believed to be significantly higher than that reported due to patients never seeking care [1]. Our results show the highest incidence of sports-related TBI in the age group 20–29 years. Given their young age, it is important to find these patients and provide them with adequate follow-up and rehabilitation.

In Sweden and especially the university city Umeå, cycling is common in all age groups. The most common traffic-related accidents involved bicycles, not cars, with a large percentage of the patients not having worn a helmet at the time of the accident. In a previous study, Styrke et al. [8] demonstrated that most traffic-related TBIs in our catchment area were bicycle accidents, causing almost 1 out of 10 concussions. An increasing number of TBIs caused by cycling has also been seen in a German study [28].

A predominance of men among TBI patients was demonstrated in all age groups except those aged over 80 years, which is believed to be related to women’s longer life expectancy. The ratio between men and women in our study was similar to that in a previous study at NUS [8], but different from those in other European studies [7,9,10,12], which demonstrated significantly higher incidences among men. The gender differences in our results show that men have a lower average age, a higher proportion of penetrating injuries, a higher frequency of admission to the ICU, and more emergency interventions. The causes of injury were more diverse in men. Women were generally older at the time of injury and the injuries were often caused by falls at home. There was no statistically significant gender difference in the number of CT scans performed, pathological findings detected, admission rate, or mortality. An interesting observation was that, although men appeared to have more severe injuries, mortality was slightly higher in women, for which reason may be the greater average age among women, combined with comorbidity.

The most common definition of mild TBI is GCS 13–15, with moderate TBI at GCS 9–12, and severe at GCS 3–8. However, GCS 13 is sometimes classified as moderate. There is considerable variation in the GCS scores considered to belong to a specific severity level [29]. Therefore, we chose to distinguish between GCS scores 15, 14, and 13, as previous studies have shown that these differ in terms of complication development and frequency of pathological CT findings [15]. In this study, we demonstrated large differences between different GCS classifications regarding injury characteristics, patient age, CT results, admission frequency, interventions performed, and mortality. The correlation between low GCS scores and morbidity in severe TBI is relatively strong. On the other hand, in minimal, mild, and even moderate TBIs, the classification is severely limited, with a need for continuous evaluation so as not to underestimate or overestimate an injury. Classification of TBI using GCS scores does not involve any pathophysiological or anatomical data and may be affected by systemic factors and drug effects impairing cerebral metabolism [30]. Another reason for a falsely low GCS score could be, for example, a deteriorating general condition in an elderly patient who seeks emergency care at night.

In most cases, mild TBI is considered relatively harmless and will not cause any emergency action, but it is much more difficult to interpret than previously thought. Despite a classification as minimal TBI without any visible symptoms or neurologic involvement, 10% of our patients had pathological CT findings and twelve of these patients subsequently required some form of emergency intervention. Our CT results are within the confidence interval of a previously published Swedish study, but we were able to demonstrate more contusions, ASDH, and tSAH [22]. This indicates that patients without visible symptoms or impaired consciousness may still have intracranial traumatic lesions. On the other hand, most patients with mild TBI did not require any type of emergency intervention. Thus, the major challenge is to find the few patients in need of urgent measures in the large patient flow. An improved classification system is needed wherein the inclusion of biomarkers can be of great value.

Overall, almost half of all TBI patients underwent a CT scan, regardless of the initial GCS classification of the injury. There were surprisingly high numbers for those with minimal TBI, where 4 out of 10 underwent a CT scan. However, the figure was significantly lower for the youngest age group, where the admission frequency was also lower compared with previously published data [31]. Our admission rate increased with increasing age. A German study of children showed an admission rate of 687 per 100,000 citizens per year, which was approximately 15% higher than our results [31]. In comparison with the German study, we had a slightly higher CT frequency, but more detectable pathological changes among children and adolescents. Significantly more abnormal CT scans were detected for the elderly compared with the younger patients, with 60% of all changes in those aged over 65 years and only 6% in the youngest age group. More pathological changes are detected if only patients with expected intracranial findings are selected. The high figures for the elderly may therefore be a result of the correct assessments to carry out scans being made. The challenge is knowing which patient needs a CT scan, without missing anyone. Selecting the right patients is also necessary to reduce radiation—one of the goals of the current Scandinavian guidelines, as 1 in 5000 CT scans will lead to cancer [21,23].

Our aim has been to investigate the total flow of TBI patients via the ER; patients who were transported directly to the ICU were therefore not included. Our results on the distribution of TBI different severities are comparable to previously published data from NUS [8], although our findings show an even higher proportion of minimal and mild TBIs and a lower level of severe injuries. Here, however, there was a major difference wherein our study cohort prospectively included all TBI patients who visited the ER. This is the second study from our region that shows a different TBI distribution than in other regions, with lower rates of severe and moderate TBIs. Previous hypotheses have argued that this can partly be explained by different injury characteristics and injury patterns [8]. Another reason, as previously discussed, may be our broader definition of TBI, particularly for minimal TBI. In another European study, mild TBI (GCS 13–15) was detected for 71–98% of patients, suggesting that our region is at the higher end of the spectrum [12]. The high flow of TBI patients in the ER represents a major challenge wherein healthcare resources need to be optimized to be used correctly.

### 4.1. Strengths and Limitations

One of the strengths of this study is that it is based on data on all TBI patients who visited the ER at NUS over a period of 24 months. We have collected large amounts of patient data without any exceptions based on age, gender, or degree of severity, thus yielding high generalizability. Our demographic and epidemiological data are representative of the area and population, as they are consistent with previously published data [8]. We also examined a large patient population that has never been described prospectively, only retrospectively [8,22]. Thus, the risk of recall bias is eliminated.

This study suffers from a few limitations. First, alcohol and drug use were not included due to too few blood samples having been taken. At our ER, ethanol use is usually analyzed with a breathalyzer without the result being recorded in the patient’s medical chart. Second, biomarkers such as S-100B were not included, also because too few analyses were performed. Third, medication with anticoagulants or other risk factors which may influence the outcome was not considered. Fourth, few patients suffer from only one isolated condition. Many of our patients had sustained multiple injuries and/or suffered from several medical conditions, along with traveling long distances to the hospital. This meant that hospitalization may, in some cases have been justified purely for practical or moral reasons; this may affect the results.

Lastly, one may speculate on whether the real incidence of TBI is even higher than what we report. We have no data on how many individuals do not visit the ER despite having suffered a head injury. Furthermore, there may be a number of patients who visit a general practitioner and thus never come to the ER. This is a problem affecting official statistics in many countries.

### 4.2. Conclusions

We have shown a high incidence of TBI in our catchment area, with large flows of patients to our ER. The challenge is carrying out the initial emergency assessment in a fast, efficient, but also accurate way without missing any patient who requires further emergency measures. Our results suggest that mild TBI may not be as harmless as previously reported due to a relatively high frequency of detected intracranial changes. Many of the TBI patients without any symptoms or neurological involvement underwent a CT scan, with only a few having detectable pathological changes. No patients in the GCS 13–15 group with a normal CT scan required any kind of emergency intervention. The majority of those that died were elderly. These findings have implications for the daily clinical practice in the acute assessment of TBI patients. To reduce the high frequency of unnecessary CT scans, a combination of biomarkers in the ER is proposed to exclude those who need a CT scan. All patients who are discharged home must receive verbal and written information on how to act if new symptoms appear and how to seek help if rehabilitation is needed. Further studies are needed to improve the initial assessment and flow at the ER of this large patient group.

## Figures and Tables

**Table 1:** Demographic and epidemiological characteristics by age group.

Category		Age group (years)													
		<20		20–29		30–49		50–64		65–79		80–99		Total	
		n%		n%		n%		n%		n%		n%		n%	
Total number		1438		**673**		531		442		489		484		4057	
Gender	Men	857	60	405	60	294	55	269	61	267	55	194	40	2286	56
	Women	581	40	268	40	237	45	173	39	222	45	290	60	1771	44
Glasgow Coma Scale score	<9	2	0.1	4	1	1	0.2	3	1	7	1	3	1	20	0.5
	9–12	1	0.1	8	1	7	1	3	1	5	1	7	1	31	1
	13	5	0.3	6	1	4	1	5	1	7	1	9	2	36	1
	14	18	1	12	2	7	1	18	4	31	6	48	10	134	3
	15	1412	98	643	96	512	96	413	93	439	90	417	86	3836	95
Pre–injury systematic disease	Normal	1202	84	581	86	378	71	182	41	93	19	41	8	2477	61
	Mild	233	16	78	12	111	21	140	32	210	43	182	38	954	24
	Moderate	3	0.2	12	2	41	8	116	26	171	35	228	47	571	14
	Severe	0	0	2	0.3	1	0.2	4	1	15	3	33	7	55	1
Injury type	Blunt/Closed	1422	99	663	99	529	100	434	98	485	99	483	100	4016	99
	Crush	15	1	3	0.4	0	0	1	0.2	0	0	0	0	25	1
	Penetrating	1	0.1	7	1	2	0.4	7	2	4	1	1	0.2	16	0.4
Injury mechanism	Fall standing height	256	18	167	25	182	34	244	55	378	77	427	88	1654	41
	Fall from height	715	50	54	8	39	7	46	10	54	11	24	5	932	23
	Bicycle accident	71	5	107	16	77	15	55	12	14	3	13	3	337	8
	Struck by or against object	144	10	60	9	57	11	33	7	13	3	11	2	318	8
	Sports injury	123	9	90	13	39	7	6	1	6	1	0	0	264	7
	Assault	52	4	112	17	55	10	21	5	4	1	0	0	244	6
	Car accident	40	3	72	11	63	12	28	6	18	4	6	1	227	6
	Other MVT accident	28	2	11	2	19	4	8	2	2	0.4	3	1	71	2
	Unclear	9	1	0	0	0	0	1	0.2	0	0	0	0	10	0.2
Place injury occurred	Home	598	42	105	16	124	23	132	30	228	47	334	69	1521	37
	Public place	105	7	203	30	131	25	167	38	177	36	75	15	858	21
	Street/Traffic	130	9	181	27	150	28	87	20	38	8	23	5	609	15
	Sports field/Horse stable	164	11	96	14	53	10	10	2	7	1	1	0	331	8
	Outside	173	12	21	3	21	4	14	3	16	3	4	1	249	6
	Work	4	0.3	38	6	33	6	22	5	3	1	0	0	100	2
	School/Kindergarten	254	18	0	0	1	0.2	1	0.2	1	0.2	0	0	257	6
	Hospital/Nursing home	1	0.1	2	0.3	3	1	2	0.5	19	4	46	10	73	2
	Others	7	0.5	17	3	5	1	0	0	0	0	1	0.2	30	1
	Unknown	2	0.1	10	1	10	2	7	2	0	0	0	0	29	1
CT scan	Performed	198	14	320	48	300	56	280	63	382	78	399	82	1879	46
	Abnormal	12	6	17	5	19	6	37	13	67	18	65	16	217	12
Admission *	Yes	353	25	112	17	111	21	114	26	205	42	255	53	1150	28
	Ward	347	98	103	92	99	89	101	89	181	88	250	98	1081	94
	ICU	6	2	9	8	12	11	13	11	24	12	5	2	69	6
Outcome	Mortality	0	0	1	0.1	0	0	0	0	5	1	16	3	22	0.5

MVT: motor vehicle and traffic accident. CT: computed tomography. ICU: intensive care unit. * Patients treated at both ICU and ward were registered once. Outcome: mortality was assessed at discharge from hospital.

**Table 2:** Demographic and epidemiological characteristics by GCS score.

Category		Glasgow Coma Scale											
		<9		9–12		13		14		15		Total	
		n%		n%		n%		n%		n%		n%	
Total number		20	0.5	31	0.8	36	0.9	134	3.3	3836	95	4057	
Gender	Men	12	60	20	65	20	56	86	64	2148	56	2286	56
	Women	8	40	11	35	16	44	48	36	1688	44	1771	44
Age group (years)	<20	2	10	1	3	5	14	18	13	1412	37	1438	35
	20–29	4	20	8	26	6	17	12	9	643	17	673	17
	30–49	1	5	7	23	4	11	7	5	512	13	531	13
	50–64	3	15	3	10	5	14	18	13	413	11	442	11
	65–79	7	35	5	16	7	19	31	23	439	11	489	12
	80–99	3	15	7	23	9	25	48	36	417	11	484	12
Pre–injury systematic disease	Normal	9	45	12	39	17	47	38	28	2401	63	2477	61
	Mild	8	40	5	16	6	17	40	30	895	23	954	24
	Moderate	3	15	14	45	13	36	45	34	496	13	571	14
	Severe	0	0	0	0	0	0	11	8	44	1	55	1
Injury type	Blunt/Closed	19	95	31	100	35	97	133	99	3804	99.2	4016	99
	Crush	0	0	0	0	0	0	0	0	19	0.5	25	0.6
	Penetrating	1	5	0	0	1	3	1	1	13	0.3	16	0.4
Injury mechanism	Fall standing height	6	30	18	58	16	44	96	72	1518	40	1654	41
	Fall from height	4	20	4	13	5	14	13	10	906	24	932	23
	Bicycle accident	4	20	4	13	3	8	9	7	317	8	337	8
	Struck by or against object	2	10	0	0	1	3	4	3	311	8	318	8
	Sports injury	0	0	0	0	3	8	4	3	257	7	264	7
	Assault	0	0	2	6	7	19	2	1	233	6	244	6
	Car accident	3	15	1	3	0	0	4	3	219	6	227	6
	Other MVT accident	1	5	2	6	0	0	1	1	67	2	71	2
	Unclear	0	0	0	0	1	3	1	1	8	0.2	10	0.2
Place injury occurred	Home	7	35	12	39	13	36	64	48	1425	37	1521	37
	Public place	3	15	9	29	6	17	24	18	816	21	858	21
	Street/Traffic	8	40	5	16	3	8	15	11	578	15	609	15
	Sports Field/Horse stable	0	0	0	0	4	11	3	2	324	8	331	8
	Outside	1	5	2	6	2	6	3	2	241	6	249	6
	Work	0	0	0	0	0	0	2	1	98	3	100	2
	School/Kindergarten	0	0	1	3	2	6	6	4	248	6	257	6
	Hospital/Nursing home	0	0	1	3	3	8	16	12	53	1	73	2
	Others	0	0	0	0	1	3	0	0	29	1	30	1
	Unknown	1	5	1	3	2	6	1	1	24	1	29	1
CT scan	Performed	19 †	95	30	97	31	86	113	84	1686	44	1879	46
	Abnormal	13	68	12	40	11	35	19	17	162	10	217	12
Admission *	Yes	19 †	95	22	71	25	69	78	58	1007	26	1151	28
	Ward	3	16	15	68	18	72	73	94	972	97	1081	94
	ICU	16	84	7	32	7	28	5	6	35	3	70	6
Outcome	Mortality	8	40	2	6	0	0	4	3	8	0.2	22	0.5

* Patients treated at both ICU and ward were registered once. † One patient was pronounced dead before the CT scan. Outcome: mortality was assessed at discharge from the hospital.

**Table 3:** Characteristics of CT results and emergency interventions.

CT Pathology	Glasgow Coma Scale Score												Gender			
	<9		9–12		13		14		15		Total		Men ♂		Women ♀	
	n%		n%		n%		n%		n%		n%		n%		n%	
Total number of patients	20	0.5	31	0.8	36	0.9	134	3.3	3836	95	4057		2286	56	1771	44
Total number of CT scan	19 †	95	30 ††	97	31	86	113	84	1686	44	1879	46	1059	46	820	46
Pathological CT scan	13	72	12	40	11	35	19	17	162	10	217	12	136	7	81	4
Compressed basal cisterns	4	31	1	8	2	18	0	0	5	3	12	6	10	7	2	2
Midlineshift-total	5	38	4	33	2	18	4	21	15	9	30	14	16	12	14	17
0–4 mm	0	0	0	0	0	0	2	11	7	4	9	4	3	2	7	9
>5 mm	0	0	1	8	2	18	1	5	3	2	7	3	9	7	2	2
>10 mm	5	38	3	25	0	0	1	5	5	3	14	6	4	3	5	6
tSAH	8	62	2	17	7	64	9	47	61	38	87	40	54	40	33	41
EDH	0	0	1	8	1	9	0	0	5	3	7	3	6	4	1	1
ASDH	7	54	5	42	4	36	8	42	48	30	72	33	43	32	29	36
Contusions	4	31	7	58	6	55	7	37	45	28	69	32	42	31	27	33
Other ^1^	2	15	1	8	1	9	3	16	35	22	42	19	28	21	14	17
Total fracture	2	15	2	17	2	18	5	26	28	17	39	18	29	21	10	12
Facial fractures	0	0	0	0	0	0	0	0	11	7	11	5	9	7	2	2
Skull fractures	2	15	1	8	2	18	5	26	16	10	26	12	18	13	8	10
Dens fractures	0	0	1	8	0	0	0	0	1	1	2	1	2	1	0	0
**Patients with Intervention**	14	70	4	13	4	11	3	2	12	0.3	37	0.9	29	1.3	8	0.5
Intubation	14	100	4	100	0	0	1	33	2	17	21	57	16	55	5	63
Decompressive craniectomy	1	7	0	0	1	25	0	0	0	0	2	5	1	3	1	13
Craniotomy evacuation of hematoma	4	29	1	25	3	75	2	67	10	83	20	54	18	62	2	25
Insertion of ICP monitoring ^2^	6	43	2	50	1	25	0	0	1	8	10	27	8	28	2	25
External ventricular drainage ^3^	2	14	1	25	1	25	0	0	1	8	5	14	4	14	1	13
Outcome-mortality	8	40	2	6	0	0	4	3	8	0.2	22	0.5	9	0.4	13	0.7

CT scan, type of pathology, and emergency intervention in numbers and percentages. The percentages are based on the total number of patients/interventions within each GCS or gender group. Each CT scan may show several pathologies and patients may have undergone multiple interventions. tSAH: traumatic subarachnoid hemorrhage. EDH: epidural hematoma. ASDH: acute subdural hematoma. ICP: intracranial pressure. ^1^ Non-traumatic or non-acute intracranial findings. ^2^ Denotes an intraparenchymal device. ^3^ Used for ICP measurement and drainage. † One patient was pronounced dead before the CT scan. †† One patient (GCS12) was hospitalized without CT scan. Outcome: mortality was assessed at discharge from the hospital.

## Data Availability

The data that support the findings of this study can be made available upon request from the corresponding author. The data are not publicly available due to ethical restrictions.
